# Comparison of postoperative deep vein thrombosis incidence between regional anesthesia with monitored anesthesia care and general anesthesia alone in total knee arthroplasty patients

**DOI:** 10.3389/fmed.2026.1800865

**Published:** 2026-04-09

**Authors:** Fang Jia, Bojun Zhang, Ping Li

**Affiliations:** Department of Anesthesia, Tianjin Hospital, Tianjin, China

**Keywords:** arthroplasty, replacement, knee, anesthesia, general, conduction, venous thrombosis, propensity score

## Abstract

**Background:**

Total knee arthroplasty (TKA) is associated with a risk of postoperative deep vein thrombosis (DVT). While regional anesthesia is integral to multimodal analgesia protocols, its specific impact on DVT risk compared to general anesthesia remains unclear.

**Methods:**

This retrospective cohort study included 250 patients undergoing unilateral TKA. Based on anesthetic records, patients were categorized into a regional anesthesia with monitored anesthesia care group (RA group, *n* = 78) or a general anesthesia alone group (GA group, *n* = 172). The RA protocol comprised spinal anesthesia and peripheral nerve blocks, with propofol titrated to maintain mild sedation and spontaneous ventilation Propensity score matching created 78 balanced pairs. The primary outcome was the incidence of in-hospital DVT, assessed via venous ultrasound. Secondary outcomes included pulmonary embolism, readmissions, ambulation time, length of stay, pain scores, opioid consumption, D-dimer levels, and complications.

**Results:**

After matching, no significant difference was found in the overall incidence of postoperative DVT or symptomatic pulmonary embolism between groups (*p* > 0.05). However, the RA group demonstrated superior secondary outcomes: significantly lower 30-day readmission rates, earlier time to first ambulation, and shorter hospital stay (*p* < 0.05). Postoperative pain scores and 24-h intravenous morphine consumption were significantly reduced in the RA group3. Plasma D-dimer levels on postoperative days 1 and 3 were also lower in the RA group (*p* < 0.05). The incidences of postoperative nausea and vomiting and pulmonary complications were significantly reduced with RA. Multivariate analysis confirmed that anesthesia type was not an independent predictor for DVT (*p* > 0.05), whereas advanced age and higher BMI were significant risk factors.

**Conclusion:**

With standardized pharmacologic prophylaxis, combined regional-general anesthesia did not further reduce the incidence of DVT after TKA compared to general anesthesia alone. Nonetheless, it provided significantly better analgesia, facilitated earlier functional recovery, shortened hospitalization, and lowered the risks of nausea, vomiting, and pulmonary complications. These findings support the inclusion of regional anesthesia as a key component for optimizing perioperative management in TKA patients.

## 1 Introduction

Total knee arthroplasty (TKA) represents a definitive surgical intervention for end-stage knee osteoarthritis, effectively restoring joint function (1–3). The annual volume of TKA procedures continues to rise, driven by an aging population and advancing surgical techniques. Despite its success, postoperative complications remain a concern, with deep vein thrombosis (DVT) and its potential sequela, pulmonary embolism (PE), constituting significant threats to perioperative morbidity and mortality (4, 5).

The pathophysiology of venous thromboembolism is classically framed by Virchow’s triad: endothelial injury, venous stasis, and hypercoagulability. Major orthopedic surgery inherently engages all three components. Surgical trauma directly damages the vascular endothelium and activates the coagulation cascade. Intraoperative tourniquet use and postoperative pain-induced limb immobilization markedly reduce lower extremity venous flow. Furthermore, the surgical stress response systemically promotes a pro-inflammatory and hypercoagulable state (6). Consequently, identifying strategies to optimize perioperative management and mitigate thrombotic risk is of paramount clinical importance.

The choice of anesthetic technique, a cornerstone of perioperative care, may influence patient recovery and complication profiles through multiple pathways. Regional anesthesia (RA) techniques, encompassing neuraxial and peripheral nerve blocks, provide superior postoperative analgesia, facilitating opioid-sparing strategies that reduce opioid-related adverse effects such as respiratory depression and gastrointestinal dysfunction (7). Beyond analgesia, RA is postulated to modulate the surgical stress response. Evidence suggests that, compared to general anesthesia (GA), RA may attenuate activation of the neuroendocrine stress axis (e.g., hypothalamic–pituitary–adrenal axis), decrease the release of stress hormones like catecholamines and cortisol, and potentially mitigate the systemic inflammatory response to surgical trauma (8). Theoretically, this blunting of physiological stress could improve perioperative microcirculation and hemodynamic stability, potentially exerting a favorable influence on thrombogenic risk (9).

Although the combined use of regional and general anesthesia (RA) is increasingly adopted in TKA to harness the benefits of both techniques, clinical evidence regarding its direct impact on postoperative DVT incidence, compared to GA alone, remains inconsistent and limited (10). Most relevant studies have focused on intermediate outcomes like analgesic efficacy, opioid consumption, or early mobility, with a relative paucity of high-quality research directly comparing DVT as a primary endpoint (10). Conclusions from some retrospective studies are challenged by inadequate control for confounding effects from baseline characteristics such as age, comorbidities, and surgical duration (11–13). Additionally, there is a lack of systematic perioperative assessment regarding the dynamic effects of different anesthetic regimens on biomarkers reflective of a hypercoagulable state, like D-dimer (14, 15). These limitations leave the precise role and value of regional anesthesia in post-TKA thromboprophylaxis incompletely defined.

To address this gap, we conducted a retrospective cohort analysis utilizing propensity score matching (PSM) to minimize confounding bias. This study systematically compares the incidence of in-hospital postoperative DVT between patients receiving RA and those receiving GA alone for TKA. Furthermore, we comprehensively evaluated differences between groups across multiple domains: thromboembolic events, early functional recovery, inflammatory and hypercoagulability biomarkers, postoperative pain control, complication profiles, and healthcare resource utilization. This investigation aims to provide more comprehensive evidence to inform clinical anesthetic strategy and explore potential underlying biological mechanisms.

## 2 Materials and methods

### 2.1 Study design and participants

This single-center, retrospective, observational cohort study reviewed electronic medical records and anesthesia clinical information systems to identify all adult patients who underwent primary, elective, unilateral TKA between January 1, 2019, and June 30, 2024. A total of 250 patients met the inclusion criteria and constituted the final analytic cohort. This study was approved by the Institutional Review Board of Tianjin Hospital (approval number: 20250511–017), and the requirement for written informed consent was waived due to the retrospective nature of the study, which involved only anonymized data. To effectively control for confounding bias while maximizing data utility, we employed propensity score matching. The caliper width for nearest-neighbor matching was determined based on the baseline characteristic distribution, aiming to achieve inter-group balance while retaining the maximal sample size.

### 2.2 Inclusion and exclusion criteria

#### 2.2.1 Inclusion criteria

1. Age ≥ 18 years;
2. Scheduled for first-time, elective, unilateral TKA;
3. American Society of Anesthesiologists (ASA) physical status I-III;
4. Availability of complete electronic anesthesia records, surgical notes, and inpatient charts.

#### 2.2.2 Exclusion criteria

1. Preoperative diagnosis of lower extremity DVT or PE confirmed by color Doppler ultrasound or clinical history;
2. Missing data exceeding 20% for the primary outcome (postoperative ultrasound results) or key covariates (e.g., body mass index, major comorbidities);
3. Conversion from a planned regional technique to GA alone due to block failure or inadequate effect;
4. Documented absolute contraindications to regional anesthesia (e.g., infection at puncture site, known severe coagulopathy with International Normalized Ratio >1.5);
5. Concurrent major non-lower extremity surgery.

### 2.3 Data sources and equipment

As a retrospective data analysis, this study involved no new patient interventions. All source data originated from standard perioperative medical devices and information systems: anesthesia workstations (Dräger Primus® or GE Aisys® series) for GA delivery; patient monitors (Philips IntelliVue® series) for continuous hemodynamic monitoring; ultrasound machines for guided nerve block placement; postoperative DVT diagnosis relied on color Doppler ultrasound systems (Philips EPIQ 7 or Siemens Acuson S3000) equipped with L5-12 MHz high-frequency linear array probes; laboratory coagulation biomarker assays were performed on a Roche Cobas® 8,000 automated analyzer.

### 2.4 Study procedures

#### 2.4.1 Data extraction and grouping

Two independent researchers, blinded to the study hypothesis, extracted data using a standardized form. Discrepancies were resolved by a senior anesthesiologist. Patients were grouped based on anesthesia records: RA group – documentation of a neuraxial (spinal) anesthetic and an ultrasound-guided single-shot or continuous peripheral nerve block (e.g., adductor canal block, femoral nerve block, sciatic nerve block, or combinations) performed pre- or post-induction of anesthesia for postoperative analgesia, combined with monitored anesthesia care (MAC) involving propofol sedation with spontaneous ventilation maintained; GA group – records indicated GA alone without any neuraxial or major peripheral nerve block.

#### 2.4.2 Anesthetic and perioperative protocol

##### 2.4.2.1 RA group

Following standard monitoring, ultrasound-guided peripheral nerve block(s) were performed under aseptic conditions prior to the induction of anesthesia. Patients were then placed in the lateral decubitus position, and spinal anesthesia was administered at the L3-4 interspace using 2.0–2.5 mL of 0.5% hyperbaric bupivacaine. Upon confirmation of a stable sensory block, monitored anesthesia care (MAC) was provided, consisting of a target-controlled infusion of propofol titrated to maintain mild sedation (Ramsay Sedation Scale 2–3), with spontaneous respiration preserved throughout the procedure.

##### 2.4.2.2 GA group

Standard balanced GA was administered using midazolam, propofol, sufentanil, and rocuronium for induction, maintained with inhaled sevoflurane and continuous remifentanil infusion, with muscle relaxants supplemented as needed.

All surgeries were performed by the same orthopedic team via a standard medial parapatellar approach. A standardized, multimodal thromboprophylaxis protocol was applied to all patients per institutional guidelines. This protocol consisted of: (1) Mechanical prophylaxis: Intermittent pneumatic compression devices were applied to the contralateral leg intraoperatively and to both lower extremities postoperatively until the patient was fully ambulatory. (2) Pharmacological prophylaxis: Subcutaneous enoxaparin (40 mg once daily) was initiated 6–8 h postoperatively, provided no significant bleeding concerns were present. Enoxaparin was continued throughout the hospital stay. Preoperative antiplatelet or anticoagulant medications were managed according to standard guidelines (e.g., antiplatelets withheld 5–7 days prior to surgery). This standardized regimen was applied uniformly, with no differences between the anesthetic groups. Postoperative analgesia was standardized to intravenous patient-controlled analgesia (PCIA) with a background infusion of sufentanil plus patient-demand boluses, routinely supplemented with tropisetron for nausea prophylaxis.

#### 2.4.3 DVT screening and diagnosis

As part of the study protocol to systematically capture asymptomatic events, all patients underwent scheduled bilateral lower extremity venous color Doppler ultrasound screening on postoperative day 3 (±1 day) and again on day 7 or the day before discharge (whichever came first). This systematic, protocol-driven screening was performed in addition to standard clinical care, which typically involves ultrasound only for symptomatic patients. All ultrasound examinations were performed by one of three dedicated vascular sonographers, each with a minimum of five years of clinical experience. These sonographers were strictly blinded to the patient’s anesthetic group allocation. A standardized scanning protocol was followed, examining the common femoral, superficial femoral, popliteal, and calf trifurcation veins. DVT was diagnosed based on the inability to fully compress the vein lumen with the transducer probe, the presence of an intraluminal echogenic thrombus, and the absence of spontaneous or augmentable color flow on Doppler imaging. To ensure diagnostic consistency, all positive findings and a random 10% sample of negative examinations were reviewed by a second, independent blinded sonographer, with any discrepancies resolved by consensus. Inter-observer reliability for the primary diagnosis of DVT was excellent (*κ* = 0.92).

#### 2.4.4 Bias control via propensity score matching (PSM)

To mitigate confounding from baseline imbalances, PSM was employed. A multivariable logistic regression model estimated each patient’s propensity to receive RA, incorporating covariates: age, sex, body mass index, ASA status, history of hypertension, diabetes, coronary artery disease, chronic kidney disease, preoperative anticoagulant/antiplatelet use, and surgical duration. Using a greedy nearest-neighbor algorithm, each RA patient was matched with a GA patient having the closest propensity score, with a caliper width set at 0.02 standard deviations of the propensity score. Post-matching balance was assessed for all covariates.

### 2.5 Outcome measures

#### 2.5.1 Primary outcome

In-hospital postoperative DVT incidence, defined as any newly detected DVT (symptomatic or asymptomatic) on scheduled bilateral venous ultrasound from surgery end until discharge.

#### 2.5.2 Secondary outcomes

1. Thromboembolic events: Symptomatic PE incidence (confirmed by CT pulmonary angiography); 30-day all-cause readmission rate.
2. Early recovery metrics: Time (hours from PACU discharge) to first assisted standing/walking; total postoperative hospital stay; rate of achieving functional mobility at discharge (defined as independent ambulation ≥30 meters with a walker).
3. Biomarkers of inflammation/hypercoagulability: Plasma D-dimer levels (mg/L FEU) measured on postoperative days 1 and 3, with peak value recorded.
4. Postoperative pain and opioid use: Worst resting pain within 24 h postoperatively assessed by Visual Analog Scale (0–10); total intravenous opioid consumption in the first 24 h, converted to intravenous morphine milligram equivalents.
5. Complication profiles: Incidence of postoperative nausea/vomiting requiring medication; postoperative pulmonary complications (new-onset pneumonia, radiologically confirmed atelectasis, or non-cardiogenic respiratory insufficiency requiring >48 h of oxygen therapy) (4); superficial/deep surgical site infection; new neurological deficits.
6. Healthcare utilization: Rate of unplanned postoperative intensive care unit admission.
7. Patient-reported outcome: Overall satisfaction with perioperative pain management assessed from nursing records prior to discharge, dichotomized as “Satisfied” (“very satisfied”/"satisfied”) vs. “Not Satisfied/Neutral” (“neutral”/"dissatisfied”).

### 2.6 Statistical analysis

Analyses were performed using R software (version 4.3.0; R Core Team, Vienna, Austria)(R Core Team (2025). R: A Language and Environment for Statistical Computing. R Foundation for Statistical Computing, Vienna, Austria. URL https://www.R-project.org/). Continuous variables were tested for normality via Shapiro–Wilk test. Normally distributed data are presented as mean ± standard deviation and compared using independent/paired t-tests (pre−/post-matching). Non-normally distributed data are presented as median (interquartile range) and compared using Mann–Whitney U/Wilcoxon signed-rank tests. Categorical variables are presented as count (percentage) and compared using Chi-square/Fisher’s exact tests (pre-matching) or McNemar’s test/conditional logistic regression (post-matching). To provide robust effect estimates, a multivariable logistic regression model adjusting for age, sex, BMI, ASA status, and key comorbidities was constructed in the full unmatched cohort to calculate the adjusted odds ratio and 95% confidence interval for the association between RA and postoperative DVT [4813]. All tests were two-tailed, with *p* < 0.05 considered significant. Data completeness was high due to the retrospective design drawing from comprehensive electronic records. Patients with missing data exceeding 20% for the primary outcome or key covariates were excluded *a priori* (see Exclusion Criteria). For the remaining covariates with a very low proportion of missing data (<2%, observed only for BMI in three cases), we performed a complete-case analysis, as the impact on the overall sample size and statistical power was deemed negligible. To verify the robustness of our primary findings, a sensitivity analysis using multiple imputation by chained equations was conducted, which yielded results consistent with the complete-case analysis (data not shown).

## 3 Results

### 3.1 Comparison of patient baseline characteristics before and after propensity score matching

Following propensity score matching, the baseline characteristics were well-balanced between the RA and GA groups. As detailed in Table 1, no statistically significant differences were observed for any covariate, including demographic factors, comorbidities, preoperative medications, and surgery-related variables such as tourniquet duration (all *p* > 0.05). Furthermore, the standardized mean differences for all covariates were less than 0.1 (data not shown), indicating negligible differences and confirming the adequacy of the matching process. These results demonstrate that the matching procedure successfully created comparable cohorts, minimizing potential confounding from measured baseline variables.

**Table 1:** Comparison of patient baseline characteristics before and after propensity score matching.

Characteristic	Before matching (*n* = 250)			After matching (*n* = 156)		
	RA group (*n* = 78)	GA group (*n* = 172)	Statistic (*p*-value)	RA group (*n* = 78)	GA group (*n* = 78)	Statistic (*p*-value)
Demographics						
Age (years), Mean±SD	65.23 ± 7.84	68.71 ± 8.92	t = −2.982 (0.003)	65.23 ± 7.84	65.87 ± 8.01	t = −0.501 (0.617)
Male, *n* (%)	28 (35.90)	55 (31.98)	χ^2^ = 0.378 (0.539)	28 (35.90)	27 (34.62)	χ^2^ = 0.030 (0.862)
BMI (kg/m^2^), Mean±SD	28.45 ± 4.12	29.01 ± 4.56	t = −0.946 (0.345)	28.45 ± 4.12	28.67 ± 4.33	t = −0.336 (0.737)
ASA status, *n* (%)						
Class II	52 (66.67)	138 (80.23)	χ^2^ = 5.857 (0.016)	52 (66.67)	49 (62.82)	χ^2^ = 0.250 (0.617)
Class III	26 (33.33)	34 (19.77)		26 (33.33)	29 (37.18)	
Comorbidities, *n* (%)						
Hypertension	45 (57.69)	89 (51.74)	χ^2^ = 0.817 (0.366)	45 (57.69)	43 (55.13)	χ^2^ = 0.103 (0.748)
Diabetes mellitus	18 (23.08)	57 (33.14)	χ^2^ = 2.595 (0.107)	18 (23.08)	17 (21.79)	χ^2^ = 0.037 (0.847)
Coronary artery disease	12 (15.38)	23 (13.37)	χ^2^ = 0.186 (0.666)	12 (15.38)	10 (12.82)	χ^2^ = 0.200 (0.655)
Chronic obstructive pulmonary disease	5 (6.41)	14 (8.14)	χ^2^ = 0.229 (0.632)	5 (6.41)	7 (8.97)	χ^2^ = 0.348 (0.555)
Chronic kidney disease	4 (5.13)	23 (13.37)	χ^2^ = 3.894 (0.048)	4 (5.13)	5 (6.41)	χ^2^ = 0.122 (0.727)
Preoperative medication, *n* (%)						
Antiplatelet agents	15 (19.23)	38 (22.09)	χ^2^ = 0.267 (0.605)	15 (19.23)	16 (20.51)	χ^2^ = 0.042 (0.838)
Anticoagulants	3 (3.85)	9 (5.23)	χ^2^ = 0.216 (0.642)	3 (3.85)	3 (3.85)	^1^
Surgery-related						
Surgical duration (min), mean±SD	98.56 ± 21.34	101.89 ± 23.67	t = −1.112 (0.267)	98.56 ± 21.34	97.92 ± 20.85	t = 0.189 (0.850)
Intraoperative blood loss (ml), median [IQR]	150 [100, 200]	150 [100, 250]	Z = -0.894 (0.371)	150 [100, 200]	150 [100, 225]	Z = -0.554 (0.579)
Intraoperative blood Transfusion, *n* (%)	2 (2.56)	7 (4.07)	χ^2^ = 0.347 (0.556)	2 (2.56)	2 (2.56)	^1^
Surgical duration (min), mean±SD	98.56 ± 21.34	101.89 ± 23.67	0.267	98.56 ± 21.34	97.92 ± 20.85	0.850
Tourniquet Duration (min), mean±SD	75.23 ± 15.67	78.41 ± 18.22	0.189	75.23 ± 15.67	74.98 ± 16.03	0.921
Intraoperative blood loss (ml), median [IQR]	150 [100, 200]	150 [100, 250]	0.371	150 [100, 200]	150 [100, 225]	0.579

^1^Statistical test not applicable due to perfect balance.

### 3.2 Comparison of primary and secondary clinical outcomes in the matched cohort

Regarding the primary clinical outcome, no statistically significant differences were observed between the two groups in the overall incidence of postoperative deep vein thrombosis (DVT) or the incidence of symptomatic pulmonary embolism (PE) (both *p* > 0.05). However, the RA group demonstrated advantages in several secondary outcomes: the 30-day readmission rate, time to first ambulation, and total postoperative hospital stay were significantly lower or shorter in the RA group compared to the GA group (*p* < 0.05). Furthermore, the rate of achieving functional mobility at discharge and satisfaction with pain management were significantly higher in the RA group (*p* < 0.05) (Table 2).

**Table 2:** Comparison of primary and secondary clinical outcomes in the matched cohort (*n* = 156).

Outcome measure	RA group (*n* = 78)	GA group (*n* = 78)	Statistic	*P*-value
Primary outcome				
Overall postoperative DVT incidence, *n* (%)	5 (6.41)	8 (10.26)	χ^2^ = 0.770	0.38
Secondary outcomes				
Symptomatic pulmonary embolism, *n* (%)	0 (0.00)	2 (2.56)	Fisher’s Exact Test	0.497
30-day readmission rate, *n* (%)	3 (3.85)	9 (11.54)	χ^2^ = 3.920	0.048
Time to first ambulation (h), median [IQR]	22.5 [18.0, 28.0]	30.0 [24.0, 36.0]	Z = -4.112	<0.001
Postoperative hospital stay (days), Mean±SD	6.23 ± 1.45	7.18 ± 1.89	t = −3.432	0.001
Functional mobility achieved at discharge, *n* (%)	71 (91.03)	62 (79.49)	χ^2^ = 4.083	0.043
Satisfaction with pain management, *n* (%)	70 (89.74)	60 (76.92)	χ^2^ = 4.500	0.034

### 3.3 Comparison of postoperative plasma D-dimer levels in the matched cohort

**Figure 1:**
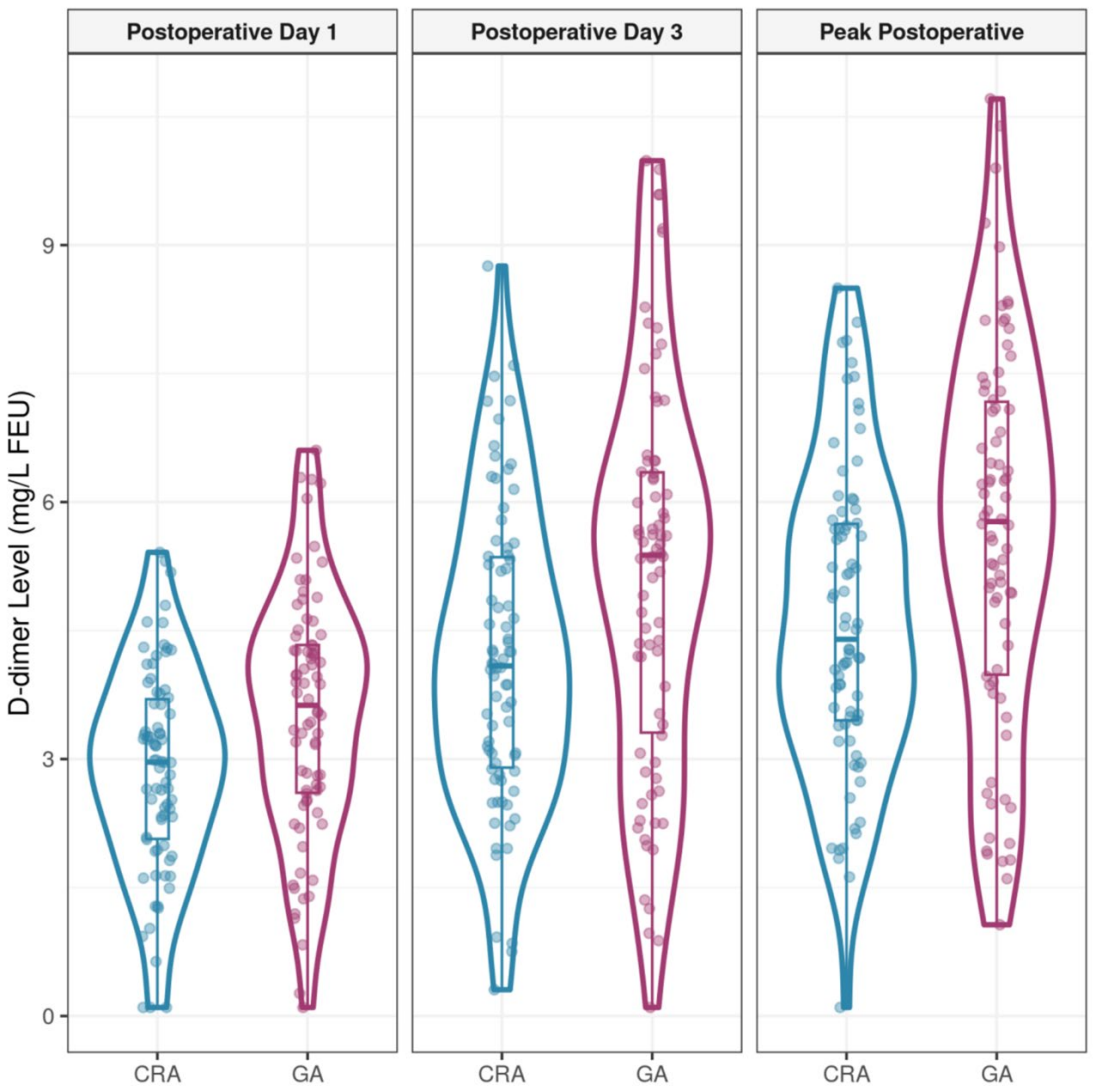
Comparison of postoperative plasma D-dimer levels in the matched cohort.

Regarding biomarkers of hypercoagulability, plasma D-dimer levels on postoperative day 1, postoperative day 3, and the peak D-dimer level were all significantly lower in the RA group compared to the GA group (all *p* < 0.05) (Table 3 and Figure 1).

**Table 3:** Comparison of postoperative plasma D-dimer levels in the matched cohort (*n* = 156).

Time point	RA group (*n* = 78), mean±SD	GA group (*n* = 78), mean±SD	*t*-value	*P*-value
Postoperative day 1 (mg/L FEU)	2.89 ± 1.23	3.67 ± 1.58	t = −3.456	0.001
Postoperative day 3 (mg/L FEU)	4.12 ± 1.87	5.34 ± 2.21	t = −3.679	<0.001
Peak postoperative D-dimer (mg/L FEU)	4.56 ± 1.92	5.89 ± 2.31	t = −3.974	<0.001

### 3.4 Comparison of postoperative pain scores and opioid consumption in the matched cohort

Regarding analgesic efficacy, patients in the RA group reported significantly lower worst resting pain scores within 24 h postoperatively. Their 24-h intravenous opioid consumption, converted to morphine milligram equivalents, was also significantly lower than that in the GA group (both *p* < 0.05) (Table 4).

**Table 4:** Comparison of postoperative pain scores and opioid consumption in the matched cohort (*n* = 156).

Measure	RA group (*n* = 78)	GA group (*n* = 78)	Statistic	*p*-value
Worst resting pain VAS score (0–10) within 24 h, mean±SD	4.23 ± 1.56	6.01 ± 1.89	t = −6.567	<0.001
24 h intravenous morphine milligram equivalents (mg), median [IQR]	15.0 [8.5, 24.0]	25.0 [18.0, 40.0]	Z = −5.234	<0.001

### 3.5 Comparison of postoperative complications and resource utilization in the matched cohort

**Figure 2:**
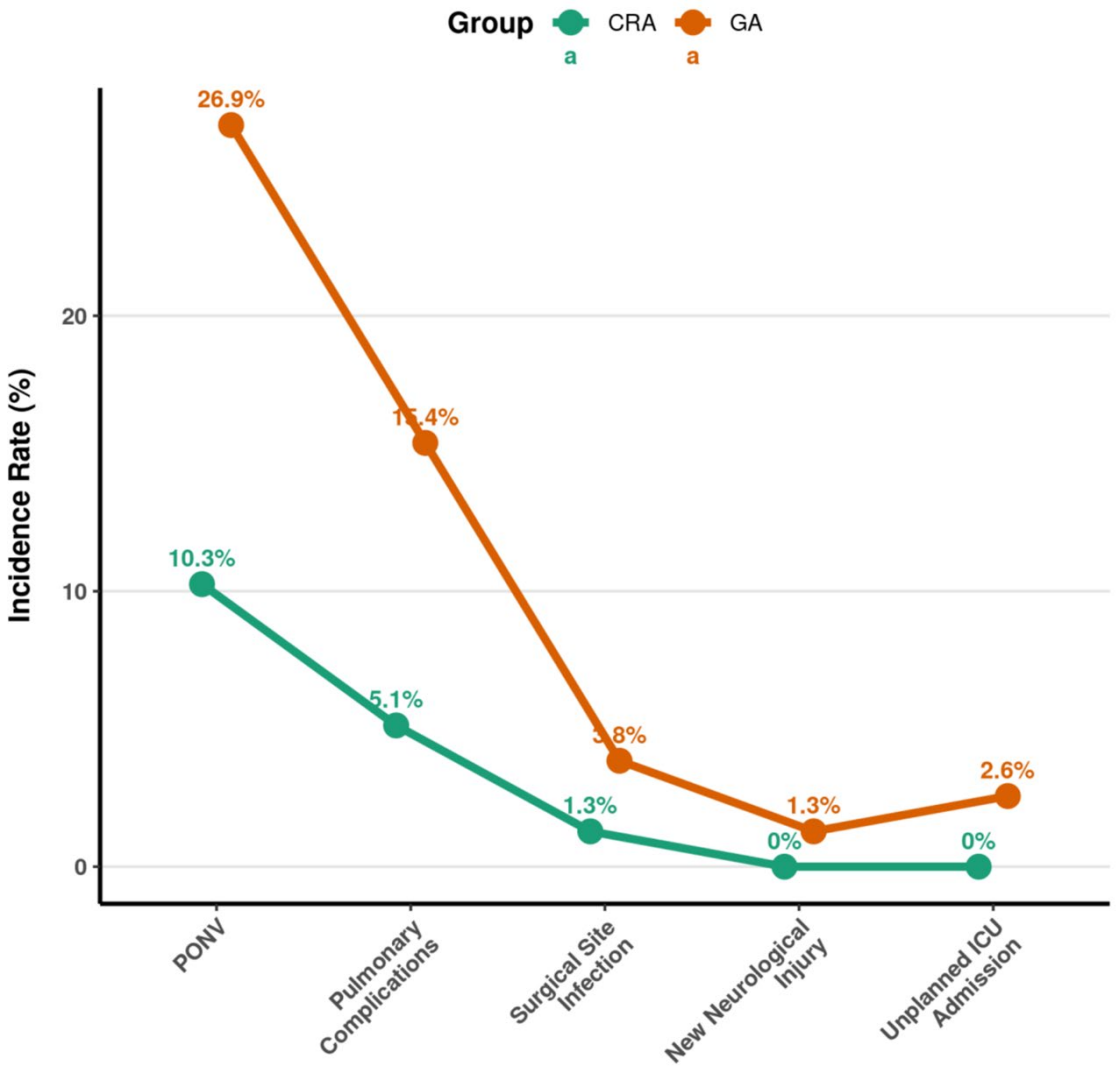
Comparison of postoperative complications and resource utilization in the matched cohort.

The incidence of postoperative nausea and vomiting (PONV) and postoperative pulmonary complications was significantly lower in the RA group compared to the GA group (both *p* < 0.05). However, there were no statistically significant differences between the groups in the rates of surgical site infection, new neurological injury, or unplanned intensive care unit (ICU) admission (all *p* > 0.05) (Table 5 and Figure 2).

**Table 5:** Comparison of postoperative complications and resource utilization in the matched cohort (*n* = 156).

Complication type	RA group (*n* = 78), *n* (%)	GA group (*n* = 78), *n* (%)	Statistic	*P*-value
Postoperative nausea and vomiting	8 (10.26)	21 (26.92)	χ^2^ = 7.143	0.008
Postoperative pulmonary complications	4 (5.13)	12 (15.38)	χ^2^ = 4.762	0.029
Surgical site infection	1 (1.28)	3 (3.85)	Fisher’s exact test	0.621
New neurological injury	0 (0.00)	1 (1.28)	Fisher’s exact test	1
Unplanned ICU admission	0 (0.00)	2 (2.56)	Fisher’s exact test	0.497

### 3.6 Multivariable logistic regression analysis for postoperative deep vein thrombosis

**Figure 3:**
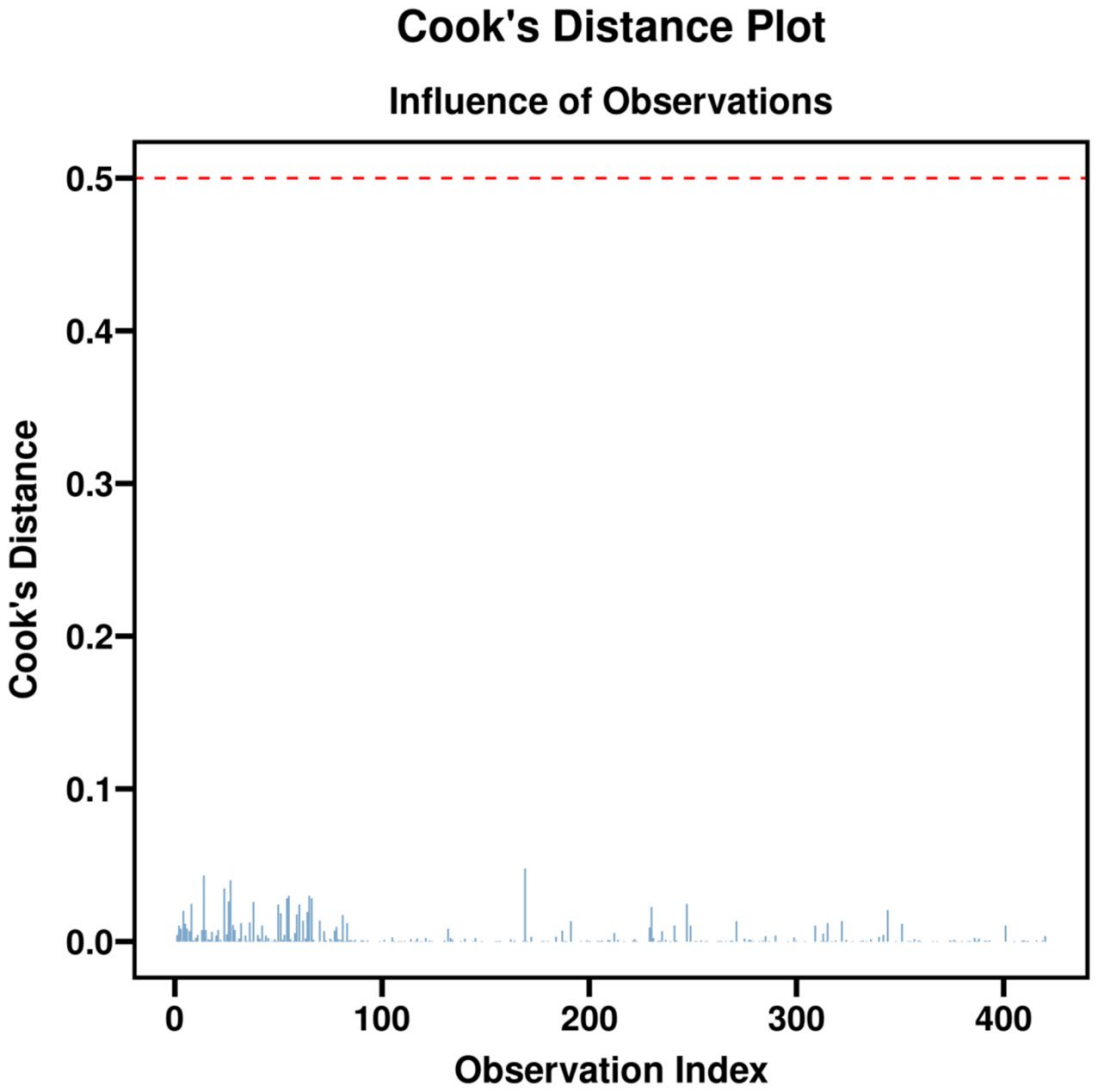
Cook’s distance plot.

**Figure 4:**
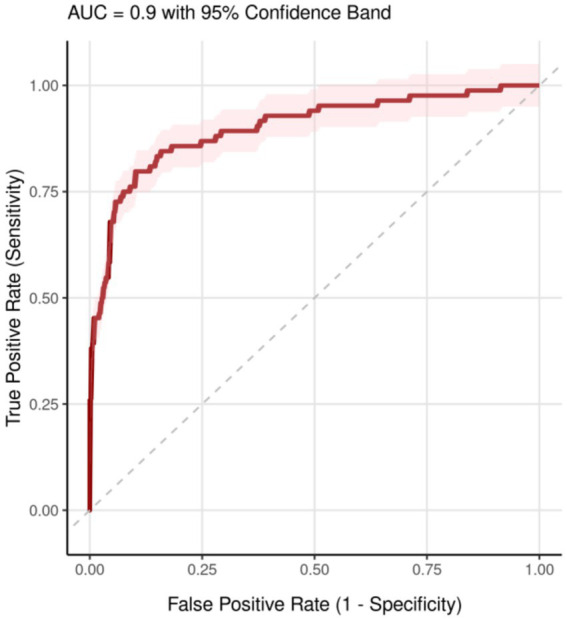
ROC curve analysis.

Multivariable logistic regression analysis of the full cohort, adjusting for age, sex, body mass index, ASA physical status, history of diabetes mellitus, and surgical duration, revealed that the anesthetic group assignment was not an independent factor associated with the occurrence of postoperative DVT (*p* > 0.05). In contrast, increasing age and higher body mass index were identified as independent risk factors for postoperative DVT (both *p* < 0.05) (Table 6 and Figures 3, 4).

**Table 6:** Multivariable logistic regression analysis for postoperative deep vein thrombosis (Full cohort, *n* = 250).

Variable	*b* value	SE	Wald χ^2^	Adjusted OR (95% CI)	*P*-value
Anesthetic group (Reference: GA group)					
RA group	−0.512	0.469	1.192	0.60 (0.24–1.50)	0.275
Age (per 1-year increase)	0.058	0.026	4.973	1.06 (1.01–1.11)	0.026
Sex (Reference: Female)					
Male	0.321	0.434	0.547	1.38 (0.59–3.23)	0.46
BMI (per 1 kg/m^2^ increase)	0.101	0.05	4.084	1.11 (1.01–1.22)	0.043
ASA status (Reference: Class II)					
Class III	0.447	0.458	0.952	1.56 (0.64–3.84)	0.329
Diabetes mellitus (Yes vs. No)	0.289	0.449	0.414	1.33 (0.55–3.22)	0.52
Surgical duration (per 10-min increase)	0.102	0.088	1.345	1.11 (0.93–1.32)	0.246

## 4 Discussion

This study aimed to investigate whether combined regional-general anesthesia (RA) offers superior postoperative recovery outcomes compared to general anesthesia (GA) alone for patients undergoing total knee arthroplasty (TKA), with a specific focus on reducing the risk of deep vein thrombosis (DVT) (16). The results demonstrated no significant difference in the overall incidence of postoperative DVT or symptomatic pulmonary embolism (PE) between the two anesthetic strategies. However, patients receiving the combined approach exhibited clear advantages across multiple secondary metrics reflecting recovery quality and perioperative safety, including reduced opioid consumption, improved early mobility, shorter hospital stays, and lower incidences of postoperative nausea and vomiting (PONV) and pulmonary complications (17). These findings suggest that while the anesthetic technique itself may not be a decisive factor influencing thrombogenesis, the adjunctive use of regional anesthesia optimizes the patient’s perioperative experience and short-term clinical outcomes through multiple pathways.

Regarding the primary outcome of DVT, the absence of a statistically significant difference between groups indicates that, in the context of standardized pharmacological thromboprophylaxis, the choice of anesthetic technique may not be a primary determinant of venous thromboembolism (VTE) incidence following TKA. Thrombosis is a complex pathological process involving endothelial injury, venous stasis, and hypercoagulability. The surgical trauma itself is a potent procoagulant stimulus, and effective perioperative anticoagulation remains the cornerstone of prevention (4). Although RA may exert some favorable hemodynamic effects by attenuating the stress response and mitigating pain-related immobilization, this effect may be insufficient to translate into an observable difference in thrombotic event rates under the dominant influence of prophylactic anticoagulation. Previous research also suggests that, within the framework of standardized thromboprophylaxis, comparisons of different anesthetic techniques on thromboembolic events after major orthopedic surgery often yield neutral results (18).

In terms of early functional recovery, patients in the RA group demonstrated earlier time to ambulation, shorter hospital length of stay, and a higher rate of achieving functional mobility at discharge. This outcome is inextricably linked to the superior postoperative analgesia provided by the combined technique. Adequate pain control is a critical prerequisite for encouraging patients to engage in early rehabilitation exercises (1). Peripheral nerve blocks, by precisely blocking nociceptive signal transmission from the surgical site, fundamentally reduce central sensitization, thereby providing analgesia in the early postoperative period that surpasses that of GA combined with intravenous analgesia alone. Reduced pain enables and motivates patients to mobilize earlier, which not only accelerates joint functional recovery but may also confer indirect benefits for thromboprophylaxis by promoting lower limb circulation (19). Regarding early functional recovery, the observed median reduction of 7.5 h in time to first ambulation (from 30.0 to 22.5 h) and the mean reduction of nearly one full day in hospital length of stay (from 7.18 to 6.23 days) represent clinically meaningful improvements. Earlier mobilization is a cornerstone of enhanced recovery protocols, potentially reducing deconditioning and the risk of other complications. A reduction in hospital stay of this magnitude has significant implications for both patient throughput and healthcare resource utilization, translating directly into cost savings and reduced exposure to nosocomial risks. Similarly, the 13.7% absolute reduction in postoperative nausea and vomiting (from 26.9 to 10.3%) is clinically substantial, as PONV is a major source of patient dissatisfaction and can delay oral intake and mobilization. The 10.2% absolute reduction in pulmonary complications, while less frequent, is equally significant given the serious nature of such events, particularly in higher-risk surgical populations.

The significantly lower peak postoperative plasma D-dimer levels observed in the RA group provide a potential biological explanation for these clinical findings. D-dimer, a fibrin degradation product, reflects the degree of activation of the coagulation and fibrinolytic systems and is a sensitive marker of a postoperative hypercoagulable state. Regional anesthesia, particularly neuraxial or major plexus blocks, has been shown to significantly attenuate the surgical stress-induced release of hormones (e.g., catecholamines, cortisol) and the cytokine storm (8). This modulation of the neuroendocrine stress response may partially mitigate the prothrombotic state triggered by surgical trauma, manifesting as lower D-dimer levels. However, whether such improvement in biochemical markers consistently translates into a reduction in hard clinical endpoints, such as thrombotic events, requires confirmation in larger-scale studies (20). However, it is important to note that while the lower D-dimer levels in the RA group (peak mean difference of 1.33 mg/L FEU) suggest a statistically significant attenuation of the hypercoagulable state, this biochemical improvement did not translate into a statistically significant or clinically detectable reduction in the primary endpoint of DVT. This dissociation underscores the dominant role of effective pharmacological thromboprophylaxis and suggests that the observed biochemical changes, while interesting, may not be of a magnitude sufficient to alter hard clinical outcomes in this setting.

The significantly reduced 24-h postoperative opioid consumption and improved pain scores observed in the RA group strongly support the analgesic efficacy of regional anesthesia. This aligns with recent studies indicating that peripheral nerve blocks are central to implementing opioid-sparing strategies in TKA. Excessive opioid use is associated with numerous adverse effects, including respiratory depression, gastrointestinal inhibition, nausea and vomiting, cognitive dysfunction, and potential opioid-induced hyperalgesia. Reducing perioperative opioid requirements through regional techniques not only enhances analgesic quality but also directly lowers the risk of complications driven by opioid-related side effects, constituting a key mechanism by which the combined approach improves overall recovery quality (21).

The lower incidence of PONV and pulmonary complications in the RA group likely results from multiple synergistic factors. First, as noted, the reduction in opioid consumption directly decreases the risk of vomiting mediated by stimulation of the chemoreceptor trigger zone. Second, regional anesthesia avoids the direct airway irritation from instrumentation (e.g., endotracheal tube) and the respiratory depressant effects of inhaled and intravenous anesthetic agents used in GA. Preserving spontaneous respiration or minimizing the impact of general anesthetics on respiratory function is particularly important for patients with pre-existing respiratory conditions (22). Previous research in thoracic and vascular surgery also suggests that regional techniques can help reduce postoperative pulmonary complications (23). The differences observed in our study corroborate that these advantages may also be realized in major orthopedic surgery.

Multivariable logistic regression analysis revealed that, after adjusting for confounders, the anesthetic technique itself was not an independent predictor of DVT occurrence. Instead, advanced age and higher body mass index were identified as independent risk factors. This finding further underscores the central role of patient-specific factors in thrombogenesis. While propensity score matching can balance observed confounding variables, it cannot eliminate all potential biases (24). This study is retrospective and observational in design. Although rigorous matching and multivariable adjustment were employed to strengthen causal inference, the possibility of residual confounding remains. For instance, the choice of anesthetic technique may be associated with unobserved factors (e.g., surgeon or anesthesiologist preference, patient motivation for rehabilitation) that could themselves influence recovery outcomes.

This study has several limitations. First, its single-center, retrospective nature necessitates caution when generalizing the conclusions. Second, despite the use of propensity score matching, the influence of unmeasured confounders cannot be entirely excluded. Third, while the use of scheduled postoperative ultrasound screening increased the detection rate of asymptomatic thrombi, the clinical significance of these findings requires interpretation in the context of long-term follow-up. Future research directions include conducting multicenter, prospective randomized controlled trials to provide higher-level evidence. Further investigation is warranted to explore the differential impact of various regional block techniques (e.g., adductor canal block vs. femoral nerve block), local anesthetic types, and dosages on outcomes. Additionally, incorporating longer-term follow-up metrics, such as chronic pain incidence, joint function scores, and patient-reported outcomes, would provide a more comprehensive assessment of the long-term benefits of anesthetic strategies.

In conclusion, for patients undergoing total knee arthroplasty with standardized thromboprophylaxis, a strategy of combined regional-general anesthesia did not further reduce the incidence of postoperative deep vein thrombosis compared to general anesthesia alone. However, the combined approach significantly improved postoperative analgesia, reduced opioid-related adverse effects, facilitated early functional recovery, and lowered the risk of postoperative pulmonary complications. These findings support the integration of regional anesthesia as a key component of multimodal analgesia and enhanced recovery after surgery (ERAS) protocols, optimizing the quality of perioperative management to indirectly create favorable conditions for patient recovery.

## Data Availability

The original contributions presented in the study are included in the article/Supplementary material, further inquiries can be directed to the corresponding author/s.
