# Supplementary material for: Comparison of postoperative deep vein thrombosis incidence between regional anesthesia with monitored anesthesia care and general anesthesia alone in total knee arthroplasty patients
Source: Front Med (Lausanne). 2026 Apr 9;13:1800865. doi: 10.3389/fmed.2026.1800865 (PMC13102675; doi:10.3389/fmed.2026.1800865)
Supplement: Supplementary file 1 [file Table_1.docx]

Supplementary Table 1. Standardized Components of Anesthetic Protocols

| Component | Regional Anesthesia  (RA) with MAC Group | General Anesthesia  (GA) Alone Group |
| --- | --- | --- |
| Neuraxial Technique | Spinal anesthesia: 2.0-2.5 ml of 0.5% hyperbaric bupivacaine at L3-4 | None |
| Peripheral Nerve Block | Ultrasound-guided single-shot block (e.g., adductor canal, femoral, sciatic, or combinations) performed pre-induction | None |
| Airway Management | Spontaneous ventilation, supplemental oxygen via facemask or nasal cannula | Endotracheal intubation with mechanical ventilation |
| Induction Agents | Not applicable (spinal block provided surgical anesthesia) | Midazolam, propofol, sufentanil, rocuronium |
| Maintenance Agents | Propofol (target-controlled infusion) titrated to mild sedation (Ramsay Sedation Scale 2-3) | Inhaled sevoflurane, continuous remifentanil infusion, with supplemental rocuronium as needed |
| Level of Consciousness | Sedated, but arousable; spontaneous breathing maintained | Unconscious, no spontaneous breathing effort |
